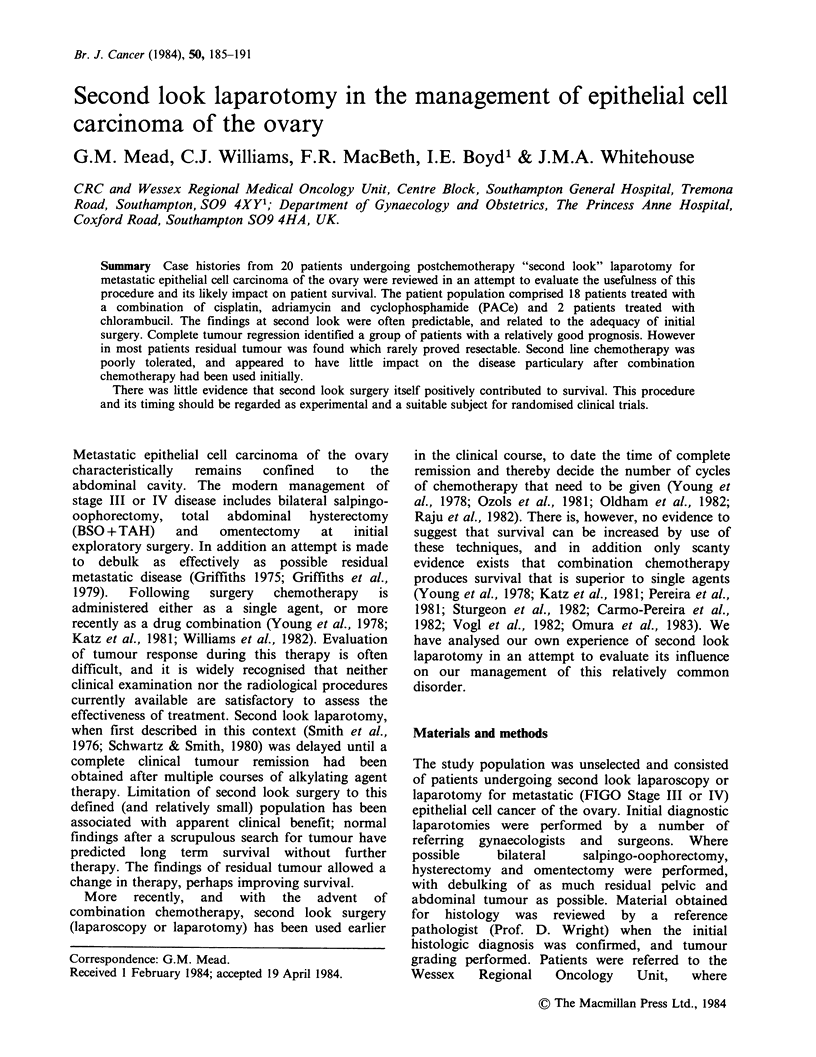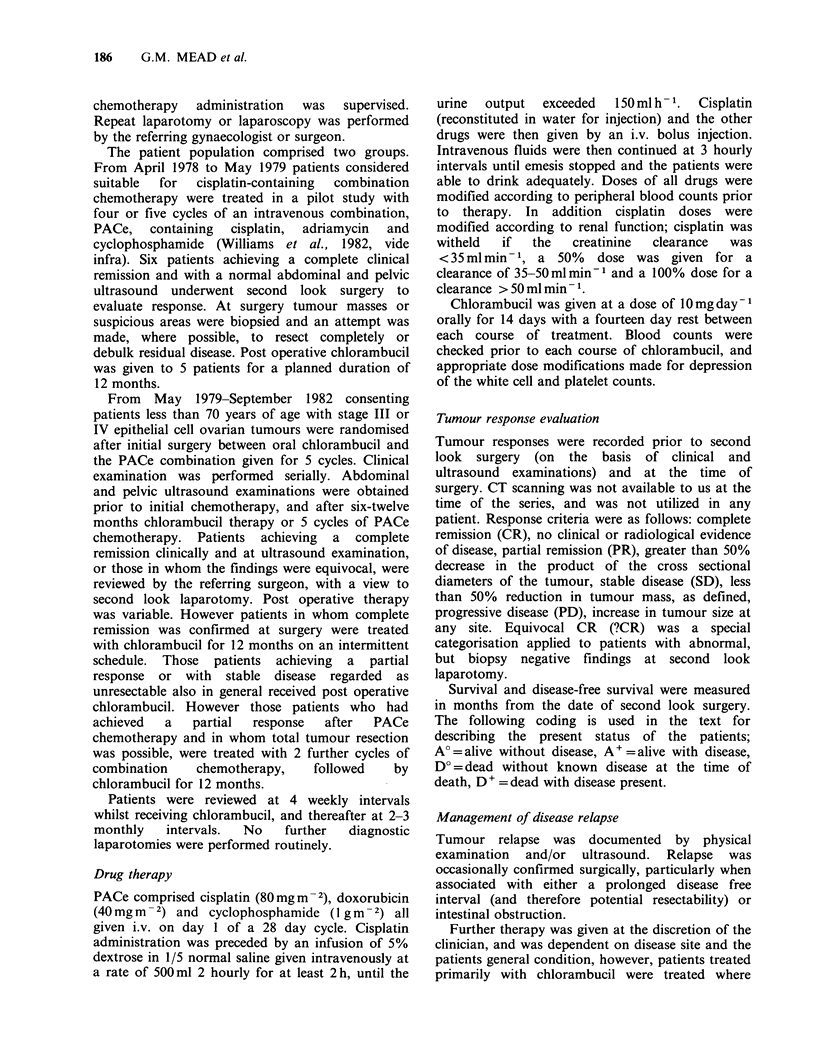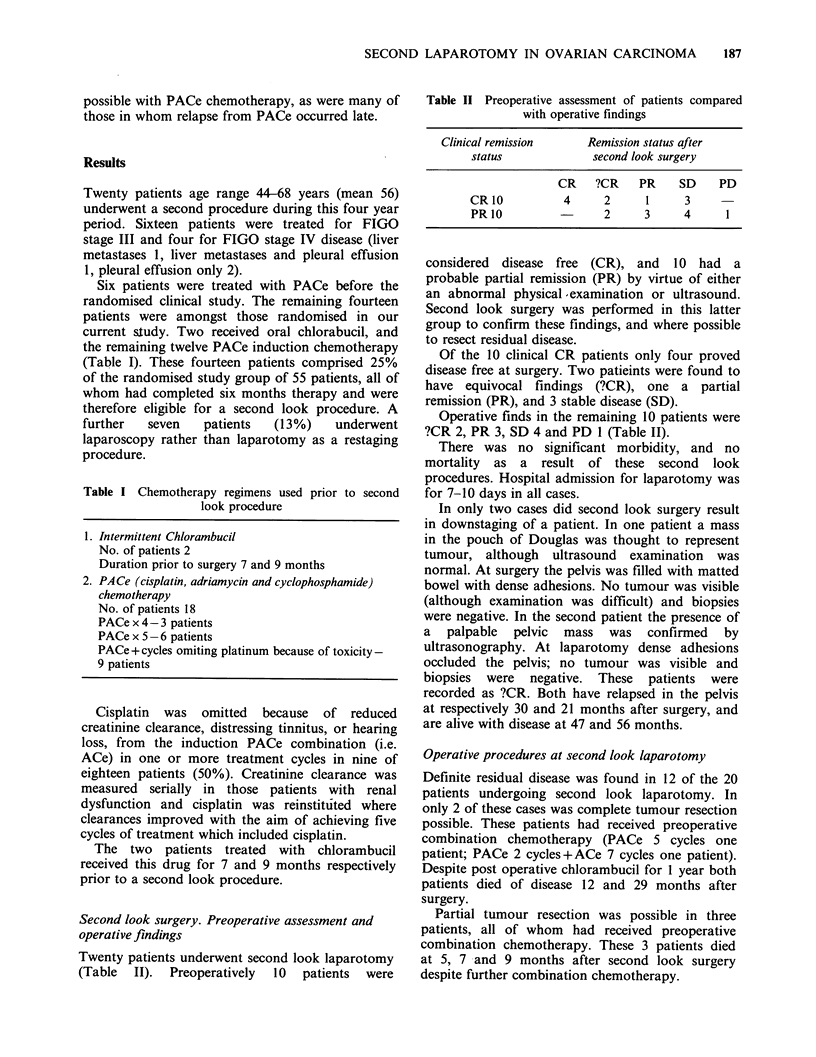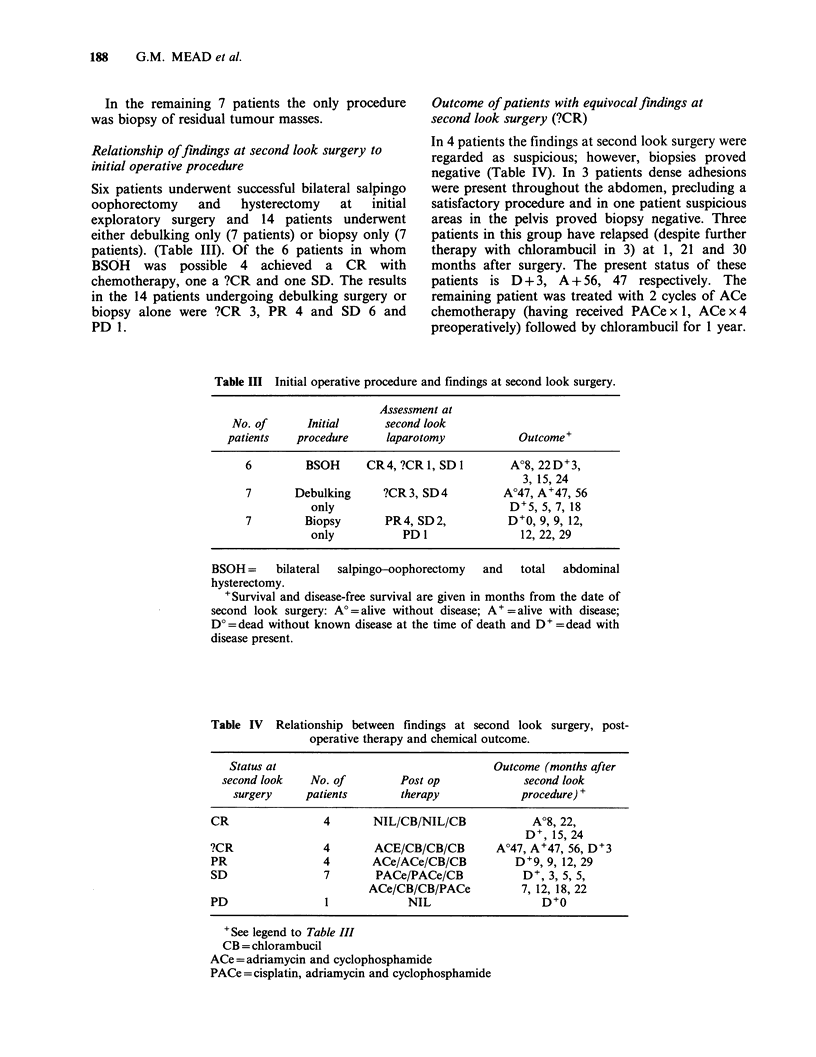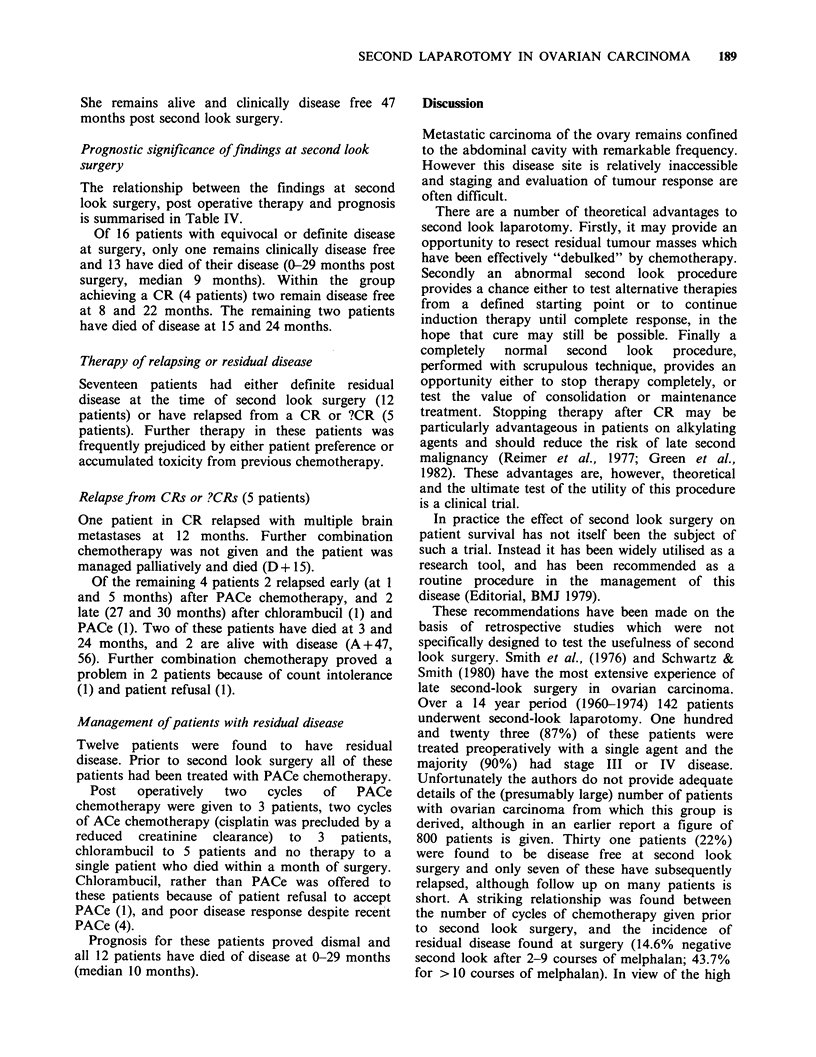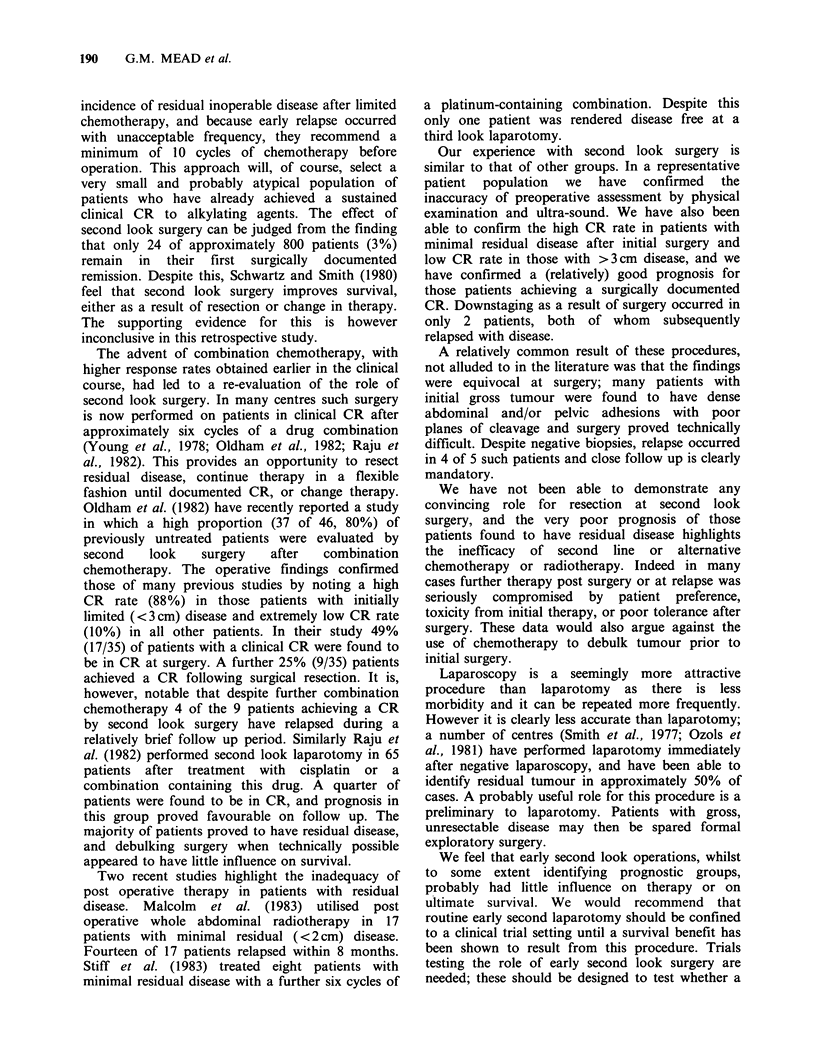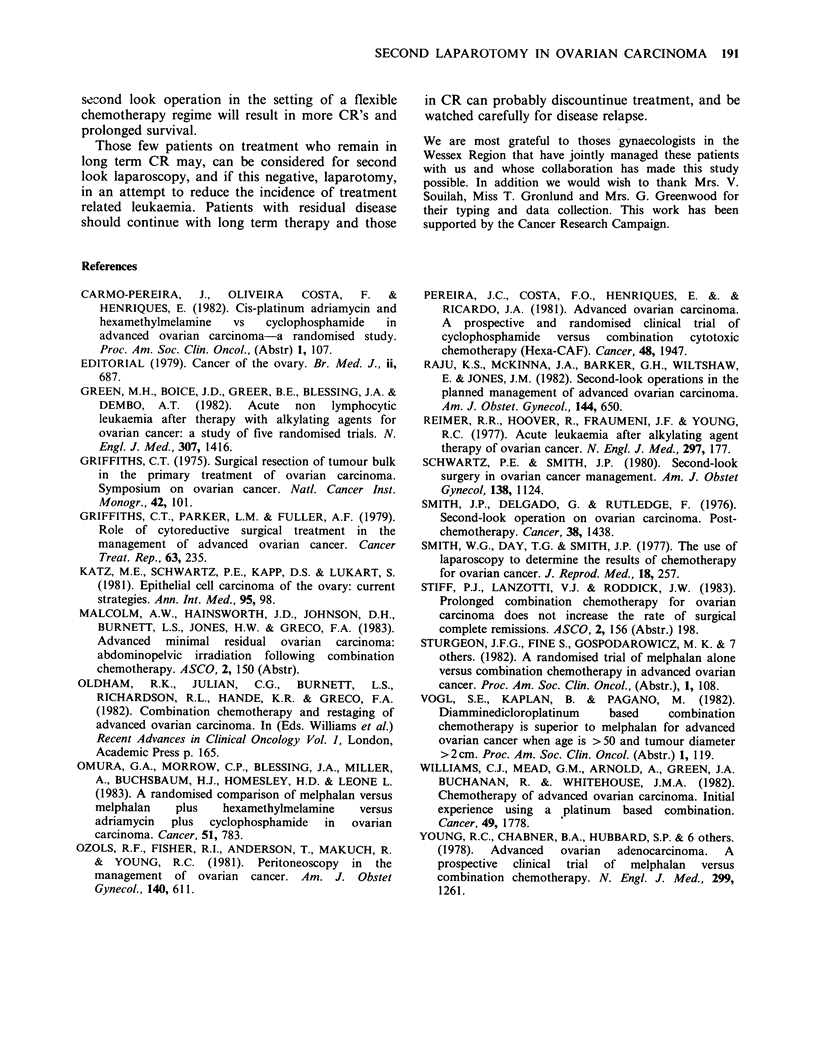# Second look laparotomy in the management of epithelial cell carcinoma of the ovary.

**DOI:** 10.1038/bjc.1984.161

**Published:** 1984-08

**Authors:** G. M. Mead, C. J. Williams, F. R. MacBeth, I. E. Boyd, J. M. Whitehouse

## Abstract

Case histories from 20 patients undergoing postchemotherapy "second look" laparotomy for metastatic epithelial cell carcinoma of the ovary were reviewed in an attempt to evaluate the usefulness of this procedure and its likely impact on patient survival. The patient population comprised 18 patients treated with a combination of cisplatin, adriamycin and cyclophosphamide (PACe) and 2 patients treated with chlorambucil. The findings at second look were often predictable, and related to the adequacy of initial surgery. Complete tumour regression identified a group of patients with a relatively good prognosis. However in most patients residual tumour was found which rarely proved resectable. Second line chemotherapy was poorly tolerated, and appeared to have little impact on the disease particularly after combination chemotherapy had been used initially. There was little evidence that second look surgery itself positively contributed to survival. This procedure and its timing should be regarded as experimental and a suitable subject for randomised clinical trials.


*Br. J. Cancer* (1984), **50**, 185–191

### Second look laparotomy in the management of epithelial cell carcinoma of the ovary

G.M. Mead, C.J. Williams, F.R. MacBeth, I.E. Boyd[1] & J.M.A. Whitehouse

*CRC and Wessex Regional Medical Oncology Unit, Centre Block, Southampton General Hospital, Tremona Road, Southampton, SO9 4XY[1]; Department of Gynaecology and Obstetrics, The Princess Anne Hospital, Coxford Road, Southampton SO9 4HA, UK.*

**Summary** Case histories from 20 patients undergoing postchemotherapy "second look" laparotomy for metastatic epithelial cell carcinoma of the ovary were reviewed in an attempt to evaluate the usefulness of this procedure and its likely impact on patient survival. The patient population comprised 18 patients treated with a combination of cisplatin, adriamycin and cyclophosphamide (PACe) and 2 patients treated with chlorambucil. The findings at second look were often predictable, and related to the adequacy of initial surgery. Complete tumour regression identified a group of patients with a relatively good prognosis. However in most patients residual tumour was found which rarely proved resectable. Second line chemotherapy was poorly tolerated, and appeared to have little impact on the disease particulary after combination chemotherapy had been used initially.

There was little evidence that second look surgery itself positively contributed to survival. This procedure and its timing should be regarded as experimental and a suitable subject for randomised clinical trials.

Metastatic epithelial cell carcinoma of the ovary characteristically remains confined to the abdominal cavity. The modern management of stage III or IV disease includes bilateral salpingo-oophorectomy, total abdominal hysterectomy (BSO+TAH) and omentectomy at initial exploratory surgery. In addition an attempt is made to debulk as effectively as possible residual metastatic disease (Griffiths 1975; Griffiths *et al.*, 1979). Following surgery chemotherapy is administered either as a single agent, or more recently as a drug combination (Young *et al.*, 1978; Katz *et al.*, 1981; Williams *et al.*, 1982). Evaluation of tumour response during this therapy is often difficult, and it is widely recognised that neither clinical examination nor the radiological procedures currently available are satisfactory to assess the effectiveness of treatment. Second look laparotomy, when first described in this context (Smith *et al.*, 1976; Schwartz & Smith, 1980) was delayed until a complete clinical tumour remission had been obtained after multiple courses of alkylating agent therapy. Limitation of second look surgery to this defined (and relatively small) population has been associated with apparent clinical benefit; normal findings after a scrupulous search for tumour have predicted long term survival without further therapy. The findings of residual tumour allowed a change in therapy, perhaps improving survival.

More recently, and with the advent of combination chemotherapy, second look surgery (laparoscopy or laparotomy) has been used earlier in the clinical course, to date the time of complete remission and thereby decide the number of cycles of chemotherapy that need to be given (Young *et al.*, 1978; Ozols *et al.*, 1981; Oldham *et al.*, 1982; Raju *et al.*, 1982). There is, however, no evidence to suggest that survival can be increased by use of these techniques, and in addition only scanty evidence exists that combination chemotherapy produces survival that is superior to single agents (Young *et al.*, 1978; Katz *et al.*, 1981; Pereira *et al.*, 1981; Sturgeon *et al.*, 1982; Carmo-Pereira *et al.*, 1982; Vogl *et al.*, 1982; Omura *et al.*, 1983). We have analysed our own experience of second look laparotomy in an attempt to evaluate its influence on our management of this relatively common disorder.

### Materials and methods

The study population was unselected and consisted of patients undergoing second look laparoscopy or laparotomy for metastatic (FIGO Stage III or IV) epithelial cell cancer of the ovary. Initial diagnostic laparotomies were performed by a number of referring gynaecologists and surgeons. Where possible bilateral salpingo-oophorectomy, hysterectomy and omentectomy were performed, with debulking of as much residual pelvic and abdominal tumour as possible. Material obtained for histology was reviewed by a reference pathologist (Prof. D. Wright) when the initial histologic diagnosis was confirmed, and tumour grading performed. Patients were referred to the Wessex Regional Oncology Unit, where

Correspondence: G.M. Mead.
Received 1 February 1984; accepted 19 April 1984.

© The Macmillan Press Ltd., 1984

chemotherapy administration was supervised. Repeat laparotomy or laparoscopy was performed by the referring gynaecologist or surgeon.

The patient population comprised two groups. From April 1978 to May 1979 patients considered suitable for cisplatin-containing combination chemotherapy were treated in a pilot study with four or five cycles of an intravenous combination, PACe, containing cisplatin, adriamycin and cyclophosphamide (Williams *et al.*, 1982, vide infra). Six patients achieving a complete clinical remission and with a normal abdominal and pelvic ultrasound underwent second look surgery to evaluate response. At surgery tumour masses or suspicious areas were biopsied and an attempt was made, where possible, to resect completely or debulk residual disease. Post operative chlorambucil was given to 5 patients for a planned duration of 12 months.

From May 1979–September 1982 consenting patients less than 70 years of age with stage III or IV epithelial cell ovarian tumours were randomised after initial surgery between oral chlorambucil and the PACe combination given for 5 cycles. Clinical examination was performed serially. Abdominal and pelvic ultrasound examinations were obtained prior to initial chemotherapy, and after six-twelve months chlorambucil therapy or 5 cycles of PACe chemotherapy. Patients achieving a complete remission clinically and at ultrasound examination, or those in whom the findings were equivocal, were reviewed by the referring surgeon, with a view to second look laparotomy. Post operative therapy was variable. However patients in whom complete remission was confirmed at surgery were treated with chlorambucil for 12 months on an intermittent schedule. Those patients achieving a partial response or with stable disease regarded as unresectable also in general received post operative chlorambucil. However those patients who had achieved a partial response after PACe chemotherapy and in whom total tumour resection was possible, were treated with 2 further cycles of combination chemotherapy, followed by chlorambucil for 12 months.

Patients were reviewed at 4 weekly intervals whilst receiving chlorambucil, and thereafter at 2–3 monthly intervals. No further diagnostic laparotomies were performed routinely.

### *Drug therapy*

PACe comprised cisplatin ($80\,mg\,m^{-2}$), doxorubicin ($40\,mg\,m^{-2}$) and cyclophosphamide ($1\,g\,m^{-2}$) all given i.v. on day 1 of a 28 day cycle. Cisplatin administration was preceded by an infusion of 5% dextrose in 1/5 normal saline given intravenously at a rate of 500 ml 2 hourly for at least 2 h, until the urine output exceeded $150\,ml\,h^{-1}$. Cisplatin (reconstituted in water for injection) and the other drugs were then given by an i.v. bolus injection. Intravenous fluids were then continued at 3 hourly intervals until emesis stopped and the patients were able to drink adequately. Doses of all drugs were modified according to peripheral blood counts prior to therapy. In addition cisplatin doses were modified according to renal function; cisplatin was witheld if the creatinine clearance was $<35\,ml\,min^{-1}$, a 50% dose was given for a clearance of $35–50\,ml\,min^{-1}$ and a 100% dose for a clearance $>50\,ml\,min^{-1}$.

Chlorambucil was given at a dose of $10\,mg\,day^{-1}$ orally for 14 days with a fourteen day rest between each course of treatment. Blood counts were checked prior to each course of chlorambucil, and appropriate dose modifications made for depression of the white cell and platelet counts.

### *Tumour response evaluation*

Tumour responses were recorded prior to second look surgery (on the basis of clinical and ultrasound examinations) and at the time of surgery. CT scanning was not available to us at the time of the series, and was not utilized in any patient. Response criteria were as follows: complete remission (CR), no clinical or radiological evidence of disease, partial remission (PR), greater than 50% decrease in the product of the cross sectional diameters of the tumour, stable disease (SD), less than 50% reduction in tumour mass, as defined, progressive disease (PD), increase in tumour size at any site. Equivocal CR (?CR) was a special categorisation applied to patients with abnormal, but biopsy negative findings at second look laparotomy.

Survival and disease-free survival were measured in months from the date of second look surgery. The following coding is used in the text for describing the present status of the patients; $A^{\circ}$ = alive without disease, $A^{+}$ = alive with disease, $D^{\circ}$ = dead without known disease at the time of death, $D^{+}$ = dead with disease present.

### *Management of disease relapse*

Tumour relapse was documented by physical examination and/or ultrasound. Relapse was occasionally confirmed surgically, particularly when associated with either a prolonged disease free interval (and therefore potential resectability) or intestinal obstruction.

Further therapy was given at the discretion of the clinician, and was dependent on disease site and the patients general condition, however, patients treated primarily with chlorambucil were treated where possible with PACe chemotherapy, as were many of those in whom relapse from PACe occurred late.

### Results

Twenty patients age range 44–68 years (mean 56) underwent a second procedure during this four year period. Sixteen patients were treated for FIGO stage III and four for FIGO stage IV disease (liver metastases 1, liver metastases and pleural effusion 1, pleural effusion only 2).

Six patients were treated with PACe before the randomised clinical study. The remaining fourteen patients were amongst those randomised in our current study. Two received oral chlorabucil, and the remaining twelve PACe induction chemotherapy (Table I). These fourteen patients comprised 25% of the randomised study group of 55 patients, all of whom had completed six months therapy and were therefore eligible for a second look procedure. A further seven patients (13%) underwent laparoscopy rather than laparotomy as a restaging procedure.

**Table I** Chemotherapy regimens used prior to second look procedure

1. *Intermittent Chlorambucil*
   No. of patients 2
   Duration prior to surgery 7 and 9 months
2. *PACe (cisplatin, adriamycin and cyclophosphamide) chemotherapy*
   No. of patients 18
   PACe x 4 – 3 patients
   PACe x 5 – 6 patients
   PACe + cycles omiting platinum because of toxicity – 9 patients

Cisplatin was omitted because of reduced creatinine clearance, distressing tinnitus, or hearing loss, from the induction PACe combination (i.e. ACe) in one or more treatment cycles in nine of eighteen patients (50%). Creatinine clearance was measured serially in those patients with renal dysfunction and cisplatin was reinstituted where clearances improved with the aim of achieving five cycles of treatment which included cisplatin.

The two patients treated with chlorambucil received this drug for 7 and 9 months respectively prior to a second look procedure.

### *Second look surgery. Preoperative assessment and operative findings*

Twenty patients underwent second look laparotomy (Table II). Preoperatively 10 patients were considered disease free (CR), and 10 had a probable partial remission (PR) by virtue of either an abnormal physical examination or ultrasound. Second look surgery was performed in this latter group to confirm these findings, and where possible to resect residual disease.

**Table II** Preoperative assessment of patients compared with operative findings

| *Clinical remission status* | *Remission status after second look surgery* | | | | |
|---|---|---|---|---|---|
| | CR | ?CR | PR | SD | PD |
| CR 10 | 4 | 2 | 1 | 3 | — |
| PR 10 | — | 2 | 3 | 4 | 1 |

Of the 10 clinical CR patients only four proved disease free at surgery. Two patieints were found to have equivocal findings (?CR), one a partial remission (PR), and 3 stable disease (SD).

Operative finds in the remaining 10 patients were ?CR 2, PR 3, SD 4 and PD 1 (Table II).

There was no significant morbidity, and no mortality as a result of these second look procedures. Hospital admission for laparotomy was for 7–10 days in all cases.

In only two cases did second look surgery result in downstaging of a patient. In one patient a mass in the pouch of Douglas was thought to represent tumour, although ultrasound examination was normal. At surgery the pelvis was filled with matted bowel with dense adhesions. No tumour was visible (although examination was difficult) and biopsies were negative. In the second patient the presence of a palpable pelvic mass was confirmed by ultrasonography. At laparotomy dense adhesions occluded the pelvis; no tumour was visible and biopsies were negative. These patients were recorded as ?CR. Both have relapsed in the pelvis at respectively 30 and 21 months after surgery, and are alive with disease at 47 and 56 months.

### *Operative procedures at second look laparotomy*

Definite residual disease was found in 12 of the 20 patients undergoing second look laparotomy. In only 2 of these cases was complete tumour resection possible. These patients had received preoperative combination chemotherapy (PACe 5 cycles one patient; PACe 2 cycles + ACe 7 cycles one patient). Despite post operative chlorambucil for 1 year both patients died of disease 12 and 29 months after surgery.

Partial tumour resection was possible in three patients, all of whom had received preoperative combination chemotherapy. These 3 patients died at 5, 7 and 9 months after second look surgery despite further combination chemotherapy.

In the remaining 7 patients the only procedure was biopsy of residual tumour masses.

*Relationship of findings at second look surgery to initial operative procedure*

Six patients underwent successful bilateral salpingo oophorectomy and hysterectomy at initial exploratory surgery and 14 patients underwent either debulking only (7 patients) or biopsy only (7 patients). (Table III). Of the 6 patients in whom BSOH was possible 4 achieved a CR with chemotherapy, one a ?CR and one SD. The results in the 14 patients undergoing debulking surgery or biopsy alone were ?CR 3, PR 4 and SD 6 and PD 1.

*Outcome of patients with equivocal findings at second look surgery* (?CR)

In 4 patients the findings at second look surgery were regarded as suspicious; however, biopsies proved negative (Table IV). In 3 patients dense adhesions were present throughout the abdomen, precluding a satisfactory procedure and in one patient suspicious areas in the pelvis proved biopsy negative. Three patients in this group have relapsed (despite further therapy with chlorambucil in 3) at 1, 21 and 30 months after surgery. The present status of these patients is D+3, A+56, 47 respectively. The remaining patient was treated with 2 cycles of ACe chemotherapy (having received PACe × 1, ACe × 4 preoperatively) followed by chlorambucil for 1 year.

**Table III** Initial operative procedure and findings at second look surgery.

| *No. of patients* | *Initial procedure* | *Assessment at second look laparotomy* | *Outcome*$^+$ |
|---|---|---|---|
| 6 | BSOH | CR 4, ?CR 1, SD 1 | A°8, 22 D$^+$3, 3, 15, 24 |
| 7 | Debulking only | ?CR 3, SD 4 | A°47, A$^+$47, 56 D$^+$5, 5, 7, 18 |
| 7 | Biopsy only | PR 4, SD 2, PD 1 | D$^+$0, 9, 9, 12, 12, 22, 29 |

BSOH = bilateral salpingo–oophorectomy and total abdominal hysterectomy.

$^+$Survival and disease-free survival are given in months from the date of second look surgery: A° = alive without disease; A$^+$ = alive with disease; D° = dead without known disease at the time of death and D$^+$ = dead with disease present.

**Table IV** Relationship between findings at second look surgery, post-operative therapy and chemical outcome.

| *Status at second look surgery* | *No. of patients* | *Post op therapy* | *Outcome (months after second look procedure)*$^+$ |
|---|---|---|---|
| CR | 4 | NIL/CB/NIL/CB | A°8, 22, D$^+$, 15, 24 |
| ?CR | 4 | ACE/CB/CB/CB | A°47, A$^+$47, 56, D$^+$3 |
| PR | 4 | ACe/ACe/CB/CB | D$^+$9, 9, 12, 29 |
| SD | 7 | PACe/PACe/CB ACe/CB/CB/PACe | D$^+$, 3, 5, 5, 7, 12, 18, 22 |
| PD | 1 | NIL | D$^+$0 |

$^+$See legend to *Table III*
CB = chlorambucil
ACe = adriamycin and cyclophosphamide
PACe = cisplatin, adriamycin and cyclophosphamide

She remains alive and clinically disease free 47 months post second look surgery.

*Prognostic significance of findings at second look surgery*

The relationship between the findings at second look surgery, post operative therapy and prognosis is summarised in Table IV.

Of 16 patients with equivocal or definite disease at surgery, only one remains clinically disease free and 13 have died of their disease (0–29 months post surgery, median 9 months). Within the group achieving a CR (4 patients) two remain disease free at 8 and 22 months. The remaining two patients have died of disease at 15 and 24 months.

*Therapy of relapsing or residual disease*

Seventeen patients had either definite residual disease at the time of second look surgery (12 patients) or have relapsed from a CR or ?CR (5 patients). Further therapy in these patients was frequently prejudiced by either patient preference or accumulated toxicity from previous chemotherapy.

*Relapse from CRs or ?CRs* (5 patients)

One patient in CR relapsed with multiple brain metastases at 12 months. Further combination chemotherapy was not given and the patient was managed palliatively and died (D + 15).

Of the remaining 4 patients 2 relapsed early (at 1 and 5 months) after PACe chemotherapy, and 2 late (27 and 30 months) after chlorambucil (1) and PACe (1). Two of these patients have died at 3 and 24 months, and 2 are alive with disease (A + 47, 56). Further combination chemotherapy proved a problem in 2 patients because of count intolerance (1) and patient refusal (1).

*Management of patients with residual disease*

Twelve patients were found to have residual disease. Prior to second look surgery all of these patients had been treated with PACe chemotherapy.

Post operatively two cycles of PACe chemotherapy were given to 3 patients, two cycles of ACe chemotherapy (cisplatin was precluded by a reduced creatinine clearance) to 3 patients, chlorambucil to 5 patients and no therapy to a single patient who died within a month of surgery. Chlorambucil, rather than PACe was offered to these patients because of patient refusal to accept PACe (1), and poor disease response despite recent PACe (4).

Prognosis for these patients proved dismal and all 12 patients have died of disease at 0–29 months (median 10 months).

### Discussion

Metastatic carcinoma of the ovary remains confined to the abdominal cavity with remarkable frequency. However this disease site is relatively inaccessible and staging and evaluation of tumour response are often difficult.

There are a number of theoretical advantages to second look laparotomy. Firstly, it may provide an opportunity to resect residual tumour masses which have been effectively "debulked" by chemotherapy. Secondly an abnormal second look procedure provides a chance either to test alternative therapies from a defined starting point or to continue induction therapy until complete response, in the hope that cure may still be possible. Finally a completely normal second look procedure, performed with scrupulous technique, provides an opportunity either to stop therapy completely, or test the value of consolidation or maintenance treatment. Stopping therapy after CR may be particularly advantageous in patients on alkylating agents and should reduce the risk of late second malignancy (Reimer *et al.*, 1977; Green *et al.*, 1982). These advantages are, however, theoretical and the ultimate test of the utility of this procedure is a clinical trial.

In practice the effect of second look surgery on patient survival has not itself been the subject of such a trial. Instead it has been widely utilised as a research tool, and has been recommended as a routine procedure in the management of this disease (Editorial, BMJ 1979).

These recommendations have been made on the basis of retrospective studies which were not specifically designed to test the usefulness of second look surgery. Smith *et al.*, (1976) and Schwartz & Smith (1980) have the most extensive experience of late second-look surgery in ovarian carcinoma. Over a 14 year period (1960–1974) 142 patients underwent second-look laparotomy. One hundred and twenty three (87%) of these patients were treated preoperatively with a single agent and the majority (90%) had stage III or IV disease. Unfortunately the authors do not provide adequate details of the (presumably large) number of patients with ovarian carcinoma from which this group is derived, although in an earlier report a figure of 800 patients is given. Thirty one patients (22%) were found to be disease free at second look surgery and only seven of these have subsequently relapsed, although follow up on many patients is short. A striking relationship was found between the number of cycles of chemotherapy given prior to second look surgery, and the incidence of residual disease found at surgery (14.6% negative second look after 2–9 courses of melphalan; 43.7% for $>10$ courses of melphalan). In view of the high incidence of residual inoperable disease after limited chemotherapy, and because early relapse occurred with unacceptable frequency, they recommend a minimum of 10 cycles of chemotherapy before operation. This approach will, of course, select a very small and probably atypical population of patients who have already achieved a sustained clinical CR to alkylating agents. The effect of second look surgery can be judged from the finding that only 24 of approximately 800 patients (3%) remain in their first surgically documented remission. Despite this, Schwartz and Smith (1980) feel that second look surgery improves survival, either as a result of resection or change in therapy. The supporting evidence for this is however inconclusive in this retrospective study.

The advent of combination chemotherapy, with higher response rates obtained earlier in the clinical course, had led to a re-evaluation of the role of second look surgery. In many centres such surgery is now performed on patients in clinical CR after approximately six cycles of a drug combination (Young *et al.*, 1978; Oldham *et al.*, 1982; Raju *et al.*, 1982). This provides an opportunity to resect residual disease, continue therapy in a flexible fashion until documented CR, or change therapy. Oldham *et al.* (1982) have recently reported a study in which a high proportion (37 of 46, 80%) of previously untreated patients were evaluated by second look surgery after combination chemotherapy. The operative findings confirmed those of many previous studies by noting a high CR rate (88%) in those patients with initially limited (<3 cm) disease and extremely low CR rate (10%) in all other patients. In their study 49% (17/35) of patients with a clinical CR were found to be in CR at surgery. A further 25% (9/35) patients achieved a CR following surgical resection. It is, however, notable that despite further combination chemotherapy 4 of the 9 patients achieving a CR by second look surgery have relapsed during a relatively brief follow up period. Similarly Raju *et al.* (1982) performed second look laparotomy in 65 patients after treatment with cisplatin or a combination containing this drug. A quarter of patients were found to be in CR, and prognosis in this group proved favourable on follow up. The majority of patients proved to have residual disease, and debulking surgery when technically possible appeared to have little influence on survival.

Two recent studies highlight the inadequacy of post operative therapy in patients with residual disease. Malcolm *et al.* (1983) utilised post operative whole abdominal radiotherapy in 17 patients with minimal residual (<2 cm) disease. Fourteen of 17 patients relapsed within 8 months. Stiff *et al.* (1983) treated eight patients with minimal residual disease with a further six cycles of a platinum-containing combination. Despite this only one patient was rendered disease free at a third look laparotomy.

Our experience with second look surgery is similar to that of other groups. In a representative patient population we have confirmed the inaccuracy of preoperative assessment by physical examination and ultra-sound. We have also been able to confirm the high CR rate in patients with minimal residual disease after initial surgery and low CR rate in those with >3 cm disease, and we have confirmed a (relatively) good prognosis for those patients achieving a surgically documented CR. Downstaging as a result of surgery occurred in only 2 patients, both of whom subsequently relapsed with disease.

A relatively common result of these procedures, not alluded to in the literature was that the findings were equivocal at surgery; many patients with initial gross tumour were found to have dense abdominal and/or pelvic adhesions with poor planes of cleavage and surgery proved technically difficult. Despite negative biopsies, relapse occurred in 4 of 5 such patients and close follow up is clearly mandatory.

We have not been able to demonstrate any convincing role for resection at second look surgery, and the very poor prognosis of those patients found to have residual disease highlights the inefficacy of second line or alternative chemotherapy or radiotherapy. Indeed in many cases further therapy post surgery or at relapse was seriously compromised by patient preference, toxicity from initial therapy, or poor tolerance after surgery. These data would also argue against the use of chemotherapy to debulk tumour prior to initial surgery.

Laparoscopy is a seemingly more attractive procedure than laparotomy as there is less morbidity and it can be repeated more frequently. However it is clearly less accurate than laparotomy; a number of centres (Smith *et al.*, 1977; Ozols *et al.*, 1981) have performed laparotomy immediately after negative laparoscopy, and have been able to identify residual tumour in approximately 50% of cases. A probably useful role for this procedure is a preliminary to laparotomy. Patients with gross, unresectable disease may then be spared formal exploratory surgery.

We feel that early second look operations, whilst to some extent identifying prognostic groups, probably had little influence on therapy or on ultimate survival. We would recommend that routine early second laparotomy should be confined to a clinical trial setting until a survival benefit has been shown to result from this procedure. Trials testing the role of early second look surgery are needed; these should be designed to test whether a second look operation in the setting of a flexible chemotherapy regime will result in more CR's and prolonged survival.

Those few patients on treatment who remain in long term CR may, can be considered for second look laparoscopy, and if this negative, laparotomy, in an attempt to reduce the incidence of treatment related leukaemia. Patients with residual disease should continue with long term therapy and those in CR can probably discountinue treatment, and be watched carefully for disease relapse.

We are most grateful to thoses gynaecologists in the Wessex Region that have jointly managed these patients with us and whose collaboration has made this study possible. In addition we would wish to thank Mrs. V. Souilah, Miss T. Gronlund and Mrs. G. Greenwood for their typing and data collection. This work has been supported by the Cancer Research Campaign.

**References**

CARMO-PEREIRA, J., OLIVEIRA COSTA, F. & HENRIQUES, E. (1982). Cis-platinum adriamycin and hexamethylmelamine vs cyclophosphamide in advanced ovarian carcinoma—a randomised study. *Proc. Am. Soc. Clin. Oncol.*, (Abstr) **1**, 107.

EDITORIAL (1979). Cancer of the ovary. *Br. Med. J.*, **ii**, 687.

GREEN, M.H., BOICE, J.D., GREER, B.E., BLESSING, J.A. & DEMBO, A.T. (1982). Acute non lymphocytic leukaemia after therapy with alkylating agents for ovarian cancer: a study of five randomised trials. *N. Engl. J. Med.*, **307**, 1416.

GRIFFITHS, C.T. (1975). Surgical resection of tumour bulk in the primary treatment of ovarian carcinoma. Symposium on ovarian cancer. *Natl. Cancer Inst. Monogr.*, **42**, 101.

GRIFFITHS, C.T., PARKER, L.M. & FULLER, A.F. (1979). Role of cytoreductive surgical treatment in the management of advanced ovarian cancer. *Cancer Treat. Rep.*, **63**, 235.

KATZ, M.E., SCHWARTZ, P.E., KAPP, D.S. & LUKART, S. (1981). Epithelial cell carcinoma of the ovary: current strategies. *Ann. Int. Med.*, **95**, 98.

MALCOLM, A.W., HAINSWORTH, J.D., JOHNSON, D.H., BURNETT, L.S., JONES, H.W. & GRECO, F.A. (1983). Advanced minimal residual ovarian carcinoma: abdominopelvic irradiation following combination chemotherapy. *ASCO*, **2**, 150 (Abstr).

OLDHAM, R.K., JULIAN, C.G., BURNETT, L.S., RICHARDSON, R.L., HANDE, K.R. & GRECO, F.A. (1982). Combination chemotherapy and restaging of advanced ovarian carcinoma. In (Eds. Williams *et al.*) *Recent Advances in Clinical Oncology Vol. 1*, London, Academic Press p. 165.

OMURA, G.A., MORROW, C.P., BLESSING, J.A., MILLER, A., BUCHSBAUM, H.J., HOMESLEY, H.D. & LEONE L. (1983). A randomised comparison of melphalan versus melphalan plus hexamethylmelamine versus adriamycin plus cyclophosphamide in ovarian carcinoma. *Cancer*, **51**, 783.

OZOLS, R.F., FISHER, R.I., ANDERSON, T., MAKUCH, R. & YOUNG, R.C. (1981). Peritoneoscopy in the management of ovarian cancer. *Am. J. Obstet Gynecol.*, **140**, 611.

PEREIRA, J.C., COSTA, F.O., HENRIQUES, E. &. & RICARDO, J.A. (1981). Advanced ovarian carcinoma. A prospective and randomised clinical trial of cyclophosphamide versus combination cytotoxic chemotherapy (Hexa-CAF). *Cancer*, **48**, 1947.

RAJU, K.S., MCKINNA, J.A., BARKER, G.H., WILTSHAW, E. & JONES, J.M. (1982). Second-look operations in the planned management of advanced ovarian carcinoma. *Am. J. Obstet. Gynecol.*, **144**, 650.

REIMER, R.R., HOOVER, R., FRAUMENI, J.F. & YOUNG, R.C. (1977). Acute leukaemia after alkylating agent therapy of ovarian cancer. *N. Engl. J. Med.*, **297**, 177.

SCHWARTZ, P.E. & SMITH, J.P. (1980). Second-look surgery in ovarian cancer management. *Am. J. Obstet Gynecol*, **138**, 1124.

SMITH, J.P., DELGADO, G. & RUTLEDGE, F. (1976). Second-look operation on ovarian carcinoma. Post-chemotherapy. *Cancer*, **38**, 1438.

SMITH, W.G., DAY, T.G. & SMITH, J.P. (1977). The use of laparoscopy to determine the results of chemotherapy for ovarian cancer. *J. Reprod. Med.*, **18**, 257.

STIFF, P.J., LANZOTTI, V.J. & RODDICK, J.W. (1983). Prolonged combination chemotherapy for ovarian carcinoma does not increase the rate of surgical complete remissions. *ASCO*, **2**, 156 (Abstr.) 198.

STURGEON, J.F.G., FINE S., GOSPODAROWICZ, M. K. & 7 others. (1982). A randomised trial of melphalan alone versus combination chemotherapy in advanced ovarian cancer. *Proc. Am. Soc. Clin. Oncol.*, (Abstr.), **1**, 108.

VOGL, S.E., KAPLAN, B. & PAGANO, M. (1982). Diamminedicloroplatinum based combination chemotherapy is superior to melphalan for advanced ovarian cancer when age is >50 and tumour diameter >2cm. *Proc. Am. Soc. Clin. Oncol.* (Abstr.) **1**, 119.

WILLIAMS, C.J., MEAD, G.M., ARNOLD, A., GREEN, J.A. BUCHANAN, R. &. WHITEHOUSE, J.M.A. (1982). Chemotherapy of advanced ovarian carcinoma. Initial experience using a platinum based combination. *Cancer*, **49**, 1778.

YOUNG, R.C., CHABNER, B.A., HUBBARD, S.P. & 6 others. (1978). Advanced ovarian adenocarcinoma. A prospective clinical trial of melphalan versus combination chemotherapy. *N. Engl. J. Med.*, **299**, 1261.